# Retinoprotective Effect of Wild Olive (Acebuche) Oil-Enriched Diet against Ocular Oxidative Stress Induced by Arterial Hypertension

**DOI:** 10.3390/antiox9090885

**Published:** 2020-09-18

**Authors:** Álvaro Santana-Garrido, Claudia Reyes-Goya, M. Carmen Pérez-Camino, Helder André, Alfonso Mate, Carmen M. Vázquez

**Affiliations:** 1 Departamento de Fisiología, Facultad de Farmacia, Universidad de Sevilla, E-41012 Sevilla, Spain; asgarrido@us.es (A.S.-G.); crgoya@us.es (C.R.-G.); vazquez@us.es (C.M.V.); 2 Epidemiología Clínica y Riesgo Cardiovascular, Instituto de Biomedicina de Sevilla (IBIS), Hospital Universitario Virgen del Rocío/Consejo Superior de Investigaciones Científicas/Universidad de Sevilla, E-41013 Sevilla, Spain; 3 Departamento de Caracterización y Calidad de lípidos, Instituto de la Grasa-CSIC, E-41013 Sevilla, Spain; mcperezcamino@ig.csic.es; 4 Department of Clinical Neuroscience, St. Erik Eye Hospital, Karolinska Institutet, 11282 Stockholm, Sweden; helder.andre@ki.se

**Keywords:** acebuche, arterial hypertension, NADPH (nicotinamide adenine dinucleotide phosphate) oxidase, nitric oxide, oxidative stress, retina, wild olive oil

## Abstract

Oxidative stress plays an important role in the pathogenesis of ocular diseases, including hypertensive eye diseases. The beneficial effects of olive oil on cardiovascular diseases might rely on minor constituents. Currently, very little is known about the chemical composition and/or therapeutic effects of the cultivated olive tree’s counterpart, wild olive (also known in Spain as acebuche—ACE). Here, we aimed to analyze the antioxidant and retinoprotective effects of ACE oil on the eye of hypertensive mice made hypertensive via administration of NG-nitro-L-arginine-methyl-ester (L-NAME), which were subjected to a dietary supplementation with either ACE oil or extra virgin olive oil (EVOO) for comparison purposes. Deep analyses of major and minor compounds present in both oils was accompanied by blood pressure monitoring, morphometric analyses, as well as different determinations of oxidative stress-related parameters in retinal layers. Aside from its antihypertensive effect, an ACE oil-enriched diet reduced NADPH (nicotinamide adenine dinucleotide phosphate) oxidase activity/gene/protein expression (with a major implication of NADPH oxidase (NOX)2 isoform) in the retinas of hypertensive mice. Supplementation with ACE oil in hypertensive animals also improved alterations in nitric oxide bioavailability and in antioxidant enzyme profile. Interestingly, our findings show that the use of ACE oil resulted in better outcomes, compared with reference EVOO, against hypertension-related oxidative retinal damage.

## 1. Introduction

Mediterranean countries account for approximately 70% of all global olive oil (OO) production, which is mainly undertaken by Spain, Turkey, Greece, Italy, Morocco, and Tunisia [1]. Not only have the beneficial effects of OO (*Olea europaea* L.) been evaluated in the context of the so-called Mediterranean diet [2] but also due to its largely recognized bioactivity [3]. Thus, the regular consumption of OO is currently associated with beneficial effects on health due to its specifically nutritional components.

A large number of physical, chemical and organoleptic characteristics is routinely used to define and classify an olive oil in different categories, following European Commission Regulation (ECC) No. 2568/91 [4]. Despite all the possible parameters to classify olive oils, the consensus is based on the maximum percentage values of free acidity, thus distinguishing between extra virgin olive oil (EVOO, ≤ 0,8%), virgin olive oil (VOO, ≤ 2%) and ordinary olive oil (OO, > 2%) [5,6].

Among the health benefits of EVOO, antioxidant, antitumoral and anti-inflammatory properties have been attributed to different components. Triacylglycerols are the main constituents of EVOO, followed by free fatty acids, glycerol, phosphatides, pigments, flavor compounds and sterols. In addition, the high proportion of unsaturated fats, mainly monounsaturated, in contrast to a low proportion of saturated fats, designs its characteristic biochemical profile [7]. In this way, oleic acid (C18:1) is the most abundant monounsaturated fatty acid (70–80%), and one of the most studied in terms of healthy effects [8]. Between 4% and 20% of polyunsaturated fatty acids (PUFAs) are represented by linoleic (C18:2) and α-linolenic (C18:3) acids, while saturated fatty acids (SFA) only account for 8–14% [9]. However, significant differences in minor components also result in diverse varieties of olive oils, which differ in quality and nutritional attributes.

Concerning minor constituents, triterpenic and phenolic compounds, tocopherols and sterols contained in EVOO have been involved in a variety of biological activities, including the activation of different signaling pathways related to redox state, homeostasis, inflammation [10,11] and epigenetics modifications of the chromatin [12,13]. As a consequence, these compounds isolated from olive oil have been recognized as powerful nutraceutical tools for the prevention and management of cardiovascular, cancer and degenerative diseases [14,15]; specifically, hydroxytyrosol (HT) and its derivates, tyrosol, oleocanthal and oleuropein, have been proven as the more remarkable compounds in this regard [16]. Other authors claim that additional minor components with still unknown bioactivity might contribute to the beneficial effects of these phenolic compounds.

The well-known PREDIMED study, a randomized controlled trial, showed the importance of EVOO in the Mediterranean diet for reducing the risk of cardiovascular disease and cardiovascular mortality, in comparison with a standard diet [17]. Moreover, neurodegenerative diseases [18] and cancer [19] presented with lower incidence in the context of the Mediterranean diet, which was in part attributed to the regular consumption of EVOO.

Unfortunately, reports on the beneficial effects of EVOO consumption against ocular diseases are still scarce. The rich fatty acid composition of ocular tissues indicates EVOO as an adequate supplement for the treatment of eye diseases. The Mediterranean diet has been linked to a lower onset and progression of age-related macular degeneration (AMD) [20,21], where EVOO might have an important role. In this sense, the Alienor study, a population study based on eye diseases in the elderly, suggested a protective role for EVOO in AMD [22], and in vitro studies showed that HT might be one of the actors responsible for this beneficial effect [23,24]. In addition, studies in rats demonstrated the neuroprotective effect of HT in the context of diabetic retinopathy (DR) [25]. Interestingly, neuroprotective properties of EVOO in DR have also been recently associated with other components of EVOO, such as oleuropein [26]. Nonetheless, the uncertain mechanisms related to the beneficial properties of EVOO consumption in ocular pathologies certainly warrants further research on this topic.

The wild olive tree (Olea europaea var. sylvestris)—also known as acebuche (ACE) when referring to the Spanish ancient specimens—is a variety of cultivated olive tree (Olea europaea var. europaea) mainly restricted to Mediterranean countries, with remarkable presence in areas such as Andalusia in Southern Spain. In spite of the copious evidence regarding the composition and beneficial effects of EVOO, very little is known about the chemical composition and/or therapeutic effects of ACE oil. Lower antigenic and allergenic capacities have been attributed to ACE in comparison with its cultivated counterpart, and preliminary studies have shown that ACE oil has a higher proportion of tocopherols (vitamin E) and sterols than EVOO [27,28].

At present, a consensus exists on the important role of oxidative stress in the pathogenesis of various systemic and retinal diseases, including AMD [29], glaucoma, retinitis pigmentosa [30] and different types of retinopathies such as DR [31] or hypertensive retinopathy [32]. In this sense, arterial hypertension (AH) has been associated with the excessive release of reactive oxygen species (ROS) through diverse molecular mechanisms, where the NADPH oxidase system and superoxide anions (O_2_^•−^) seem to be the pivotal agents [33,34]. Seven isoforms of the NADPH oxidase (NOX) system (namely NOX1-5 and Duox1-2) have been characterized so far, although the predominant NOXes in vascular cells with the highest relevance in AH development are NOX1, 2, 4 and 5 [35].

Nitric oxide (NO) metabolism is closely related to the NADPH system because excess O_2_^•−^ production can induce uncoupling of endothelial nitric oxide synthase (eNOS); this might result in endothelial dysfunction and neovascularization eventually, since NO helps maintain ocular hemodynamics by protecting the endothelial cells of vascular beds and nerve fibers against pathogenic processes, e.g., diabetes and glaucoma [36,37]. Considering that NO is a key mediator in blood pressure regulation and that NO deficiency results in AH, it seems plausible that this system participates in the development of oxidative imbalance in hypertensive retinas. However, and despite the reported relationship between AH and retinal damage [38], the precise mechanisms involved in this regard remain unclear.

The aim of this study was to explore the beneficial, antioxidant effects of a diet enriched with ACE oil, based upon its capacity to counteract ocular (retina/choroid) damage, in a rodent model of AH induced with NG-nitro-L-arginine-methyl-ester (L-NAME). To this end, blood pressure monitoring and morphometric analyses were carried out in hypertensive mice under ACE oil- or EVOO-enriched diets (for comparison purposes). Determinations of oxidative stress-related parameters in ocular layers included: estimation of reactive oxygen species (ROS) levels by dihydroethidium fluorescence; H_2_O_2_, nitrotyrosine and NO levels; activity, gene/protein expression and immunohistofluorescence of NADPH oxidase isoforms; eNOS activation and eNOS/inducible (iNOS)/arginase 1-2 expression; and quantification of antioxidant enzymes. In addition, glial fibrillary acidic protein (GFAP, as an oxidative/inflammatory marker of gliosis) and transcription factors nuclear factor kappa-B (NF-kB) and nuclear factor erythroid-2 (Nrf-2) (related to oxidative stress pathways) were also quantified.

## 2. Materials and Methods

### 2.1. Study Design

The experimental design was conducted in accordance with the European Union (EU) Directive 2010/63/EU and the national (RD 53/2013) guidelines for the care and use of laboratory animals, and was approved by the competent Institutional Animal Care and Use Committee (approval reference #13/03/2019/031, issued by Junta de Andalucía, Dirección General de Producción Agrícola y Ganadería). Male C57B/6J mice aged 10–12 weeks were obtained from the Center for Animal Production and Experimentation at the University of Seville (Spain). Mice were randomly assigned into six groups of 12 animals each: (1) normotensive mice fed a commercial diet (control group), (2) normotensive mice fed a commercial diet supplemented with 12% (w/w) of wild olive oil (ACE group), (3) normotensive mice supplemented with 12% of extra virgin olive oil (EVOO group), (4) hypertensive mice (via administration of 45 mg L-NAME/kg body weight/day) fed a standard pellet diet (L-NAME group), (5) L-NAME-induced hypertensive mice supplemented with 12% of ACE oil (LN+ACE group); and (6) L-NAME-induced hypertensive mice supplemented with 12% of EVOO (LN+EVOO group). All treatments were maintained for six weeks; during this period, food and water intake were continuously monitored. All animals were housed in a regulated environment under standard conditions (23 ± 1 °C, 12 h/12 h light/dark cycles). Upon harvesting, animal samples were collected as described below and assigned to different experiments, as specified in Figure captions.

### 2.2. Dietary Supplementation

Animals were fed a dietary pellet composition (ROD14IRR, Sodispan Research, Altromin, Germany) supplemented, where applicable, with 12% of ACE oil or EVOO, as specified in Section 2.1. The chemical composition of ACE oil and EVOO, including major and minor constituents, is detailed in Table 1.

To prepare the animal feed, pellets were crushed in powder form, then mixed with the corresponding oils, as appropriate, up to a final 12% (w/w) of oil content, ensuring a homogenate oil spread in the powder. This powder-oil mixture was used to create new feed pellets, which were kept fresh and light protected until use. ACE oil and EVOO were obtained from the same geographic area (Sierra de las Nieves, Málaga, Spain) and subjected to similar extraction methods from the corresponding fruits, according to the standard protocols to comply with extra virgin oil definition. In turn, the concentration of L-NAME in the drinking water was weekly calculated considering the evolution of body weight and water intake, and the dosage was chosen in agreement with previous studies in rodents carried out in our laboratory.

### 2.3. Determination of ACE Oil and EVOO Chemical Composition

ACE oil and EVOO composition were determined as previously reported [39,40]. Fatty acids and tocopherols were determined according to the International Union of Pure and Applied Chemistry (IUPAC) Standard Methods 2.301 and 2.432, respectively. Phenols were determined, starting from a 2.5 g oil sample. The phenol fraction was isolated by solid-phase extraction (SPE) using a diol-phase cartridge, and the extract was analyzed by reversed-phase high performance liquid chromatography (HPLC; Hewlett Packard 1050 series pumping component, Agilent Technologies, Waldbronn, Germany) coupled with diode array UV detection (RF-10AXL Shimadzu fluorescence detector, Shimadzu, Kyoto, Japan). To determine triterpenic acids, the acidic fraction of the olive oils was isolated by solid-phase extraction using bonded aminopropyl cartridges, and the extract was silylated and analyzed by gas chromatography. Sterols and triterpenic dialcohol fractions were isolated from the unsaponifiable matter by thin-layer chromatography on a basic silica gel plate, transformed into trimethylsilyl ethers and analyzed by capillary column gas chromatography (EU Reg. 2019/1604) “Official Journal of the European Union. Commission Delegated Regulation (EU) No 2019/1604 of 27 September 2019 amending Regulation (EEC) No 2568/91 on the Characteristics of Olive Oil and Olive-Residue Oil and on the Relevant Methods of Analysis; Official Journal of the European Union: Brussels, Belgium, 2019; Volume L250, pp. 14–48”. The chemical analysis of ACE and EVO oil extracts was performed using three samples from each oil type, and each datum came from triplicate measurements.

### 2.4. Animal Characteristics

Weight gain and food/water intake were registered on a daily basis. Systolic and diastolic blood pressures (BP) were measured weekly by the indirect method of tail–cuff occlusion in conscious animals using a Niprem 645 pressure recorder (CIBERTEC, Barcelona, Spain). BP values were calculated as the average of three to four successive measurements.

### 2.5. Histomorphometric Studies

Paraffin sections of 5 μm were obtained following administration of 4% paraformaldehyde (PFA) in phosphate-buffered saline (PBS) by intravitreal injection; then, eyes were post-fixed in 4% PFA for 24h. These sections were used for morphometric analysis, dihydroethidium (DHE) staining and immunohistochemistry, as described below. For morphometric analysis, images of hematoxylin/eosin-stained sections were acquired using an Olympus BX41 microscope coupled to an Olympus DP73 camera. The thickness of retinal layers was measured as previously described [41], using ImageJ-NIH freeware (v. 2.0.0) (https://imagej.nih.gov/).

### 2.6. Tissue Isolation and Homogenization

Mice were deeply anesthetized with a mix of ketamine (75 mg/Kg i.p.) and diazepam (10 mg mg/Kg i.p.) and euthanized by cervical dislocation. Retinas and retinal pigment epithelium (RPE)/choroid complexes were rapidly dissected under a binocular stereoscopic microscope, snap-frozen in liquid nitrogen and stored at −80 °C until use for molecular analyses. Both retina and RPE/choroid homogenates were obtained in 50 mM PBS (pH 7.4) with protease inhibitors (Sigma Aldrich-Roche, Madrid, Spain) using a Potter–Elvehjem tissue grinder. Homogenates were centrifuged for 10 min at 10,000× *g* and the supernatants were recovered to determine the protein concentration by the Bradford method [42].

### 2.7. NADPH Oxidase Activity Measurements

NADPH oxidase activity was measured both in retina and RPE/choroid homogenates by lucigenin-enhanced chemiluminescence, following routine protocols in our laboratory [43]. To confirm the source(s) of superoxide anion (O_2_^•−^), homogenate samples were preincubated for 5 min at 37 °C with the following inhibitors at 0.1 mmol/L: diphenyleneiodonium, DPI (inhibitor of flavoproteins; Sigma-Aldrich, Madrid, Spain); oxypurinol (inhibitor of xanthine oxidase; Sigma-Aldrich, Madrid, Spain); and rotenone (mitochondrial chain inhibitor of electron transport; Sigma-Aldrich, Madrid, Spain). Following the same protocol, the inhibitor of NOX1/4 (0.1 µmol/L GKT136901; Sigma-Aldrich, Madrid, Spain, 492000), specific NOX1 inhibitor (0.5 µmol/L ML171; Sigma-Aldrich, Madrid, Spain, 175226) and the pan-NADPH oxidase inhibitor (10 µmol/L VAS2870; Sigma-Aldrich, Madrid, Spain, 5340320001) were used to explore the relative contribution of each NOX isoform in O_2_^•−^ production [35]. Hydrogen peroxide (H_2_O_2_) levels were measured in retina homogenates by Amplex^TM^ Red hydrogen peroxide/peroxidase assay kit (A22188, ThermoFisher Scientific, Invitrogen, Spain) following the manufacturer’s instructions. Absorbance readings were obtained in 96-well plates at 560 nm. All measurements referred to the samples’ protein content, and results were always expressed as relative to the control group.

### 2.8. Retinal and Choroidal ROS Measurement

Paraffin sections (5 μm) were used to measure retinal and choroidal ROS production using a fluorescent dye with dihydroethidium (DHE; MedChemExpress, Madrid, Spain, Cat. No. HY-D0079), as described Sasaki et al., 2010 [44]. To confirm the specificity of DHE staining, eye slides were preincubated with 100 U/mL polyethylene glycol-conjugated superoxide dismutase (PEG-SOD; Sigma Aldrich, S9549) for 30 min at 37 °C. Following the same protocol, specific NOX inhibitors VAS2870, GKT136901 and ML171 were preincubated in retinal sections. 4′,6-diamidino-2-phenylindole (DAPI) Fluoromount-G^®^ (SouternBiotech Associates, Inc, Birmingham, AL; Cat. No. 0100-20) was used to mount deparaffinized sections incubated with DHE for 20 min at 37 °C. An Olympus DP73 fluorescence microscope (Tokyo, Japan) and Image J-NIH freeware (v. 2.0.0) were used to measure the intensity of the staining. The results were expressed as relative to the control group.

### 2.9. Immunohistofluorescence

The localization and expression of NOX isoforms and GFAP were evaluated by immunohistofluorescence staining on the retina and choroid in deparaffinized eye sections. Antigen retrieval compound Diva Decloaker (Biocare Medical, LLC, Pacheco, CA, USA) and primary antibodies listed in Table 2 were used for immunostaining. Goat anti-rabbit Alexa Fluor^®^ 555 (Cohesion Biosciences Ltd., London, UK; Cat. No. CSA3411), goat anti-rabbit Alexa Fluor^®^ 488 (Cat. No. CSA3211) and goat anti-mouse Alexa Fluor^®^ 647 (Cat. No. CSA3808) were used as fluorescent secondary antibodies, where appropriate, and sections were mounted with DAPI Fluoromount-G^®^.

### 2.10. Western Blotting Analyses

Aliquots of retinal homogenates containing equal amounts of proteins (40 μg) were mixed with sample buffer, subjected to sodium dodecyl sulfate—polyacrylamide gel electrophoresis (SDS-PAGE) electrophoresis and immunoblotted with specific antibodies listed in Table 3, as previously described [45]. Quantitative analysis was performed by optical densitometry (Cytiva Europe GmbH, Barcelona, Spain) using β-actin as a loading control in the same membranes.

### 2.11. Real-Time PCR

Following the TRIzol^®^ RNA isolation method (Thermo Fisher Scientific, Madrid, Spain) in retina samples, a reverse transcription reaction was performed as previously described [46]; specific primers (listed in Table 4) were then used for the amplification of gene products in a CFX96 real-time PCR system (Bio-Rad, Madrid, Spain). Glyceraldehyde-3-phosphate dehydrogenase (GAPDH) was used as a housekeeping gene to quantify the relative changes in mRNA expression following the 2^−^^ΔΔCt^ method [47].

### 2.12. Nitric Oxide (NO) Concentration

NO concentration in retina homogenates was estimated from nitrite and nitrate (NOx) levels by the Griess method [48]. NO concentrations were normalized to the protein content of each sample, and results were expressed as relative to NO concentration in the control group.

### 2.13. Statistical Analyses

All results are presented as means ± SEM. One-way ANOVA followed by a post-hoc Tukey’s multiple comparison test were performed with GraphPad InStat Software (San Diego, CA, USA, v. 3.10), and differences were considered statistically different at *p* < 0.05. Based on the stability of the values of the variables considered in this study, each one of the samples is sufficiently representative of the population of the group to which it belongs. Accordingly, the application of the Central Limit Theorem guarantees the non-violability of the hypotheses prior to the application of the ANOVA and post-hoc tests for the comparison of means.

## 3. Results

### 3.1. Oil Composition Analyses

Overall, the fatty acid composition of ACE oil was similar to that of EVOO, although the former had a higher content of palmitic (C16:0) and palmitoleic acid (C16:1; see Table 1). The acidity of ACE oil (measured as percentage of oleic acid) falls within the category of extra virgin oils. Interestingly, the quantification of minor compounds from unsaponifiable fractions showed important differences between ACE oil and EVOO. Thus, ACE oil seems richer in total sterols and tocopherols. No remarkable changes were observed in the profile of sterols except for a higher content of Stigmasterol and Δ-5,24-Stigmastadienol in ACE oil. In turn, α-tocopherol was enhanced in ACE oil, whereas the proportion of β-tocopherol was lower than that of EVOO. The proportion of triterpene acids in ACE oil (~340 mg/kg) doubled that of EVOO (~150 mg/kg), the former showing an increase in oleanolic and maslinic acids together with a decrease in ursolic acid. The proportion of triterpene alcohols was also higher in wild olive oil. Surprisingly, although total phenol content remained the same, both oils presented a completely different profile in this regard because secoiridoids were predominant in ACE oil, while EVOO was enriched in ortodiphenols. These findings might contribute to the knowledge about the healthy properties of these oils, attributable to their chemical composition.

### 3.2. Characterization of the Experimental Model

**Figure 1:**
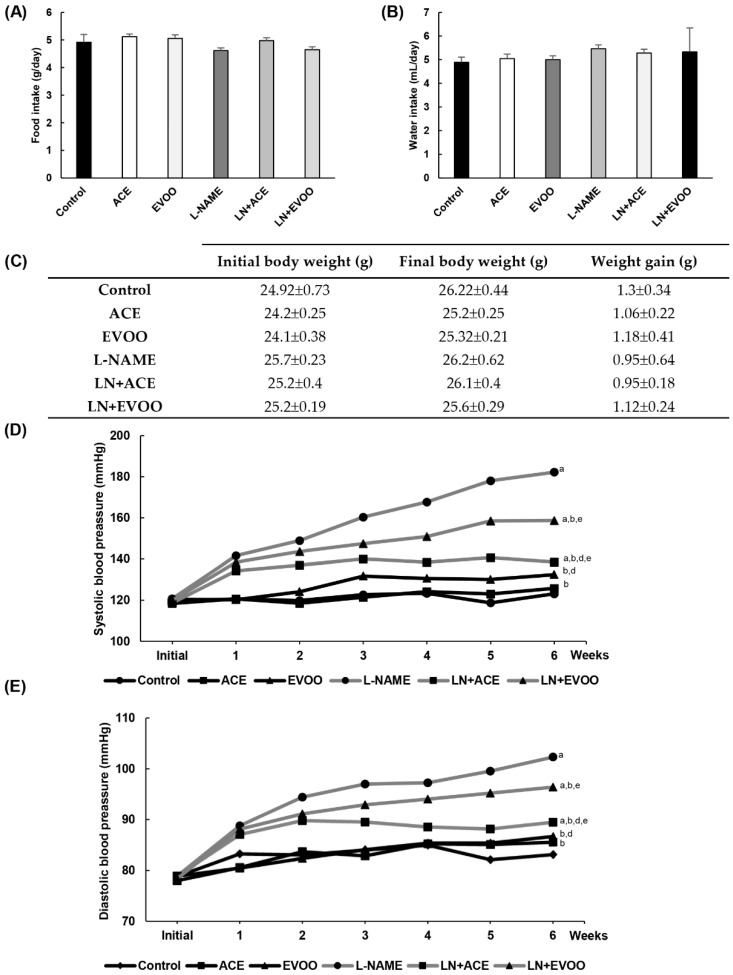
Animal general characteristics. (**A**) Food intake, (**B**) water intake and (**C**) weight gain in experimental animal groups. The weekly progression of (**D**) systolic blood pressure (SBP) and (**E**) diastolic blood pressure (DBP) are represented for each animal group. Values are expressed as mean ± SEM of seven animals per group: a *p* < 0.05 vs. control; b *p* < 0.05 vs. NG-nitro-L-arginine-methyl-ester (L-NAME); d *p* < 0.05 vs. L-NAME-induced hypertensive mice supplemented with 12% of EVOO (LN+EVOO); e *p* < 0.05 vs. ACE.

No significant changes were observed in food intake, water intake and weight gain among the study groups (Figure 1A–C). Figure 1D,E shows the evolution of systolic blood pressure (SBP) and diastolic blood pressure (DBP) values throughout the 6-week experimental period. The LN+ACE group counteracted the typical increase in blood pressure caused by NO depletion, specially from the third week onwards. On the other hand, the LN+EVOO group showed a significantly lower ability to decrease blood pressure in comparison with LN+ACE. Thus, at the end of treatment, both SBP and DBP revealed signs of marked hypertension in the L-NAME group (182/102 mmHg, respectively) when compared with the control group (123/83 mmHg). Endpoint values in the LN+ACE group were 138/89 mmHg, whereas the equivalent values for LN+EVOO were 158/96 mmHg. As for the effects of oil administration alone, the ACE group stayed with normotensive SBP/DBP values (126/86 mmHg), as did the EVOO group (132.5/86.7 mmHg).

### 3.3. Histomorphometric Effects of Dietary Supplementation

**Figure 2:**
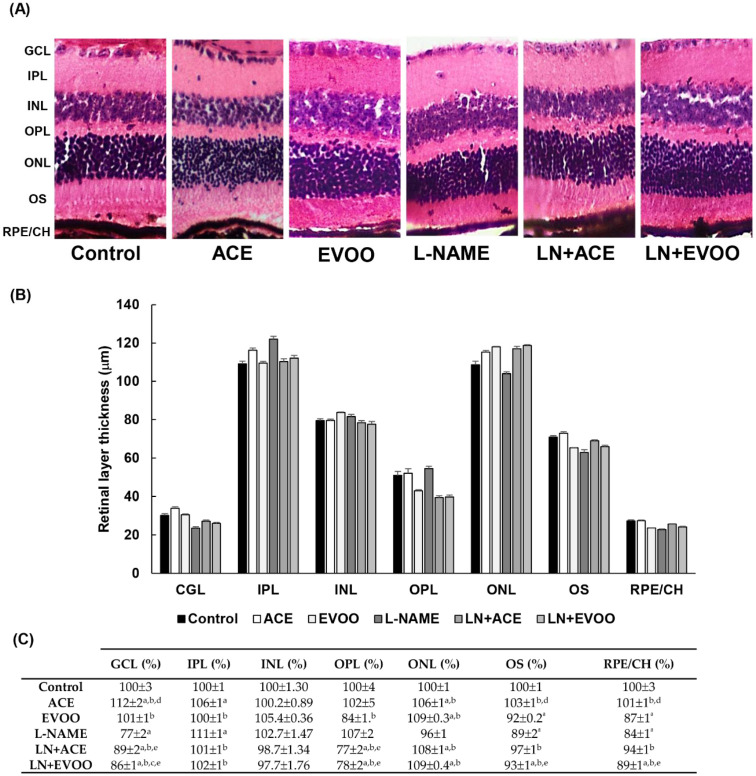
Histomorphometric analyses of retina slides. (**A**) Representative images of hematoxylin/eosin staining; (**B**) retinal layer thickness; and (**C**) percentages of thickness relative to that in the control group. Magnification: 10×. Values are expressed as mean ± SEM of seven animals per group: ^a^ *p* < 0.05 vs. control; ^b^ *p* < 0.05 vs. L-NAME; ^c^ *p* < 0.05 vs. EVOO; ^d^ *p* < 0.05 vs. LN+EVOO; ^e^ *p* < 0.05 vs. ACE. GCL: ganglion cell layer; IPL, inner plexiform layer; INL, inner nuclear layer; OPL, outer plexiform layer; ONL, outer nuclear layer; OS, outer segments; RPE/CH, retinal pigmentary epithelium/choroid.

Representative images of H/E-stained retinas are shown in Figure 2A. Overall, there were no histological differences due to dietary supplementation with ACE oil or EVOO, or secondary to treatment with L-NAME, because all groups showed normal distribution and morphology in both retinal and choroidal layers. However, a thorough morphometric analysis of retinal layer thickness (Figure 2B,C) revealed significant reductions of 22%, 11% and 16% in the ganglion cell layer (GCL), outer segments (OS) and retinal pigmentary epithelium/choroid (RPE/CH), respectively, together with a 12% increase in the inner plexiform layer (IPL) in the L-NAME group in comparison with the control group; these alterations were partially reversed when diets were supplemented with either ACE oil or EVOO. Dietary supplementation with ACE oil or EVOO in L-NAME-treated mice also resulted in the narrowing of the outer plexiform layer (OPL), and the opposite was true for the outer nuclear layer (ONL). The thickness of the inner nuclear layer (INL) remained unchanged in all groups.

### 3.4. ROS Levels and Oxidative Stress Markers in Retina

**Figure 3:**
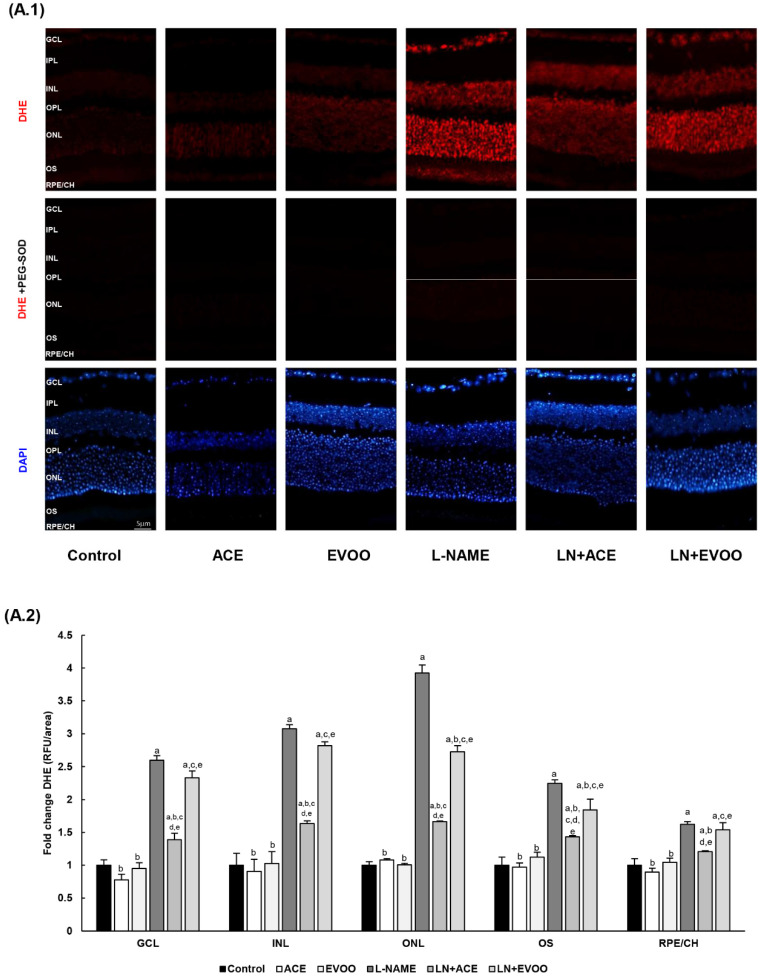

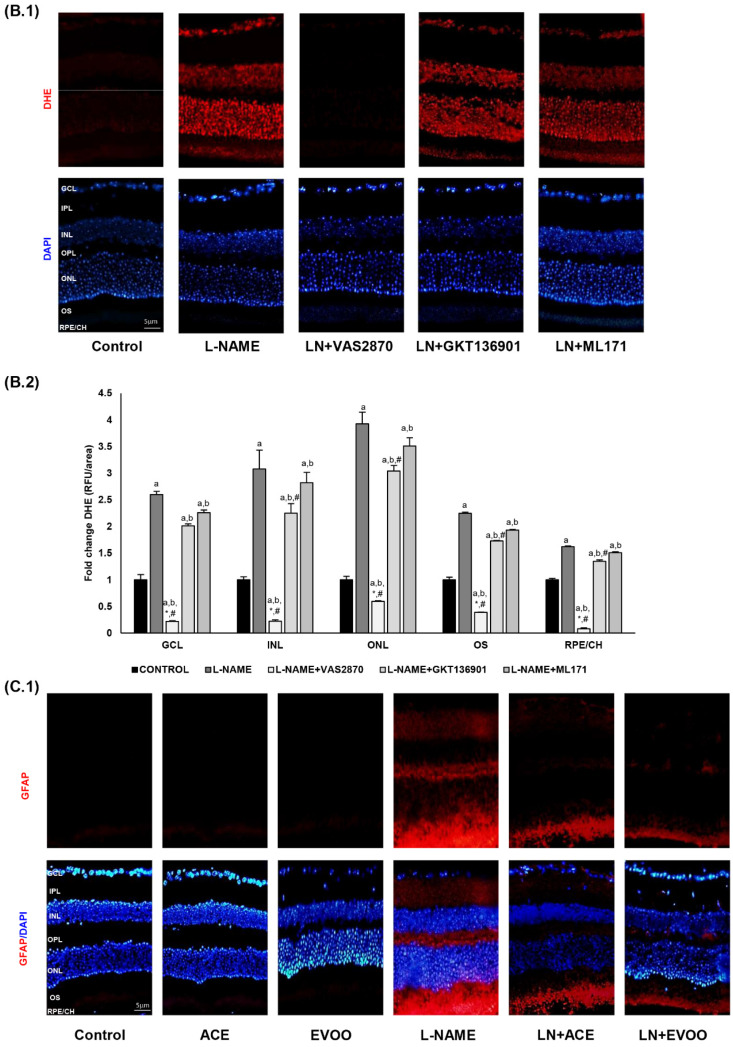

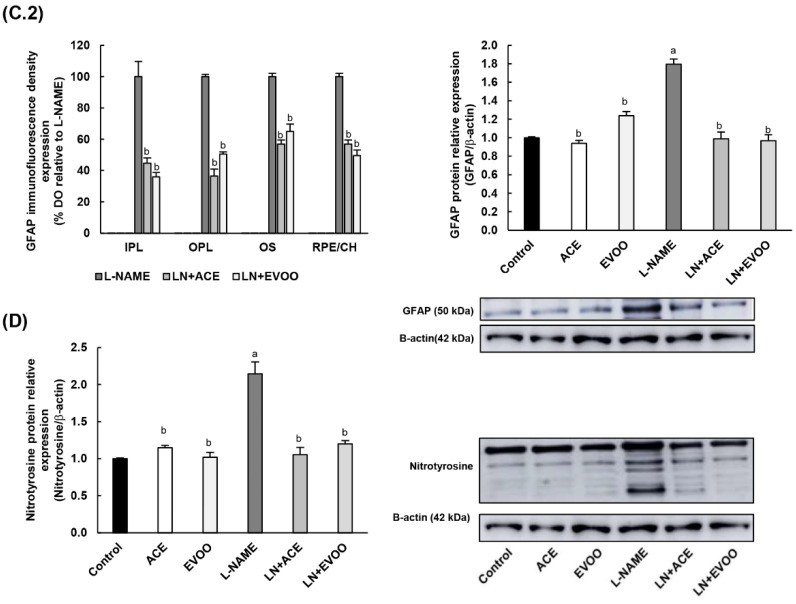
(**A.1**) Dihydroethidium (DHE) labeling (red color) for reactive oxygen species (ROS) was present in GCL, INL, ONL and OS in the retina, and also in RPE/CH layers, which can be distinguished with 4′,6-diamidino-2-phenylindole (DAPI, blue color) nuclei staining. Middle line photos in the panel represent the effects of preincubation with polyethylene glycol-conjugated superoxide dismutase (PEG-SOD). (**A.2**) Fluorescence intensity in (**A.1**) relative to that of control group and quantified using Image J software. (**B.1**) DHE labeling in retinas from L-NAME-treated mice following preincubation with specific nicotinamide adenine dinucleotide phosphate (NADPH) oxidase (NOX) inhibitors: NOX pan-inhibitor (VAS2870), dual NOX1/NOX4 inhibitor (GKT136901) and NOX1 inhibitor (ML171). (**B.2**) Relative fluorescence intensity in (**B.1**) relative to that of control group and quantified using the Image J software. (**C.1**) Localization/expression of GFAP in retinal layers, with further quantification by measurement of fluorescence intensity and by Western blotting of retinal homogenates (**C.2**). (**D**) Nitrosylation of proteins estimated by Western blotting in retinal homogenates. Magnification: 10×. Values are expressed as mean ± SEM of four animals per group: ^a^ *p* < 0.05 vs. control; ^b^ *p* < 0.05 vs. L-NAME; ^c^ *p* < 0.05 vs. EVOO; ^d^ *p* < 0.05 vs. LN+EVOO; ^e^ *p* < 0.05 vs. ACE; * *p* < 0.05 vs. LN+GKT136901; # *p* < 0.05 vs. LN+ML171. RFU, relative fluorescence units. GCL, ganglion cell layer; IPL, inner plexiform layer; INL, inner nuclear layer; OPL, outer plexiform layer; ONL, outer nuclear layer; OS, outer segments; RPE/CH, retinal pigmentary epithelium/choroid.

DHE staining showed an intensified signal in L-NAME hypertensive mice compared with the control group at all levels (2.59-, 3.08-, 3.92-, 2.25- and 1.62-fold change in GCL, INL, ONL, OS and RPE/CH, respectively), an alteration that was reversed in hypertensive animals fed with the ACE oil-enriched diet (Figure 3A.1,A.2). On the contrary, the beneficial action of EVOO in this regard was not that evident. In fact, no significant differences were found in GCL, INL and RPE/CH when comparing LN+EVOO and L-NAME groups. The effectiveness of the ACE oil supplementary diet was clearly highlighted in the LN+ACE group, where DHE staining decreased in GCL (121%), INL (144%), ONL (226%), OS (81%) and RPE/CH (42%) in comparison with L-NAME-treated animals. In turn, the LN+EVOO group showed only significant differences in comparison with the L-NAME group in ONL and OS, with respective signal reductions of 120% and 41%. The administration of oil diets to normotensive animals did not affect DHE staining. In any case, the presence of PEG-SOD (Figure 3A.1) resulted in the abolition of O_2_^•−^ production, thus confirming the specificity of DHE staining.

To clarify the specific roles of NOX isoforms in O_2_^•−.^ production, retina slides were preincubated with different NOX inhibitors (Figure 3B.1,B.2). Interestingly, our results showed the greatest reduction in DHE-dependent O_2_^•−.^ signal (~89%) when incubating retinal slides from the L-NAME group with the NOX pan-inhibitor VAS2870, whereas mild reductions were attributable to NOX1/NOX4 inhibitor GKT136901 (~22%), and specific NOX1 inhibitor ML171 (~11%). This suggests a major implication of the NOX2 isoform in terms of excessive O_2_^•−.^ generation in L-NAME hypertensive animals. No changes were observed in retinal slides from the control group in the presence and absence of these inhibitors (data not shown).

After confirming the overproduction of O_2_^•−^ in retinal layers of hypertensive mice, glial fibrillary acidic protein (GFAP) was also measured by immunofluorescence as an oxidative stress/inflammatory marker of gliosis (Figure 3C.1). GFAP-specific signals could be detected in the retinal plexiform layers (IPL, OPL), OS and RPE/CH of L-NAME-treated animals, and the staining was significantly lower in LN+ACE and LN+EVOO groups. Specifically, GFAP expression dropped to 55% (IPL), 36% (OPL), 57% (OS) and 57% (RPE/CH) in the LN+ACE group, and respective values for the LN+EVOO group were 36%, 50%, 65% and 50% (Figure 3C.2). No fluorescence signal for GFAP was apparent in the control, ACE and EVOO groups (Figure 3C.1). The quantification of GFAP protein expression in retina homogenates by Western blotting revealed an upregulation (1.8-fold) in hypertensive animals that could be prevented by oil-enriched diets (Figure 3C.2).

As an additional marker of oxidative stress, the degree of 3-nitrosylation proteins was also measured in retina homogenates by Western blotting (Figure 3D). A significant increase in this parameter was observed in the L-NAME group (2.1-fold change over all other experimental groups). Taken together, all these results suggest an imbalance in the oxidative and inflammatory processes in retinas and RPE/CH from hypertensive mice, which were partly reversed by ACE oil- and EVOO-based diets.

### 3.5. NADPH Oxidase Activity in Retina and Choroid

**Figure 4:**
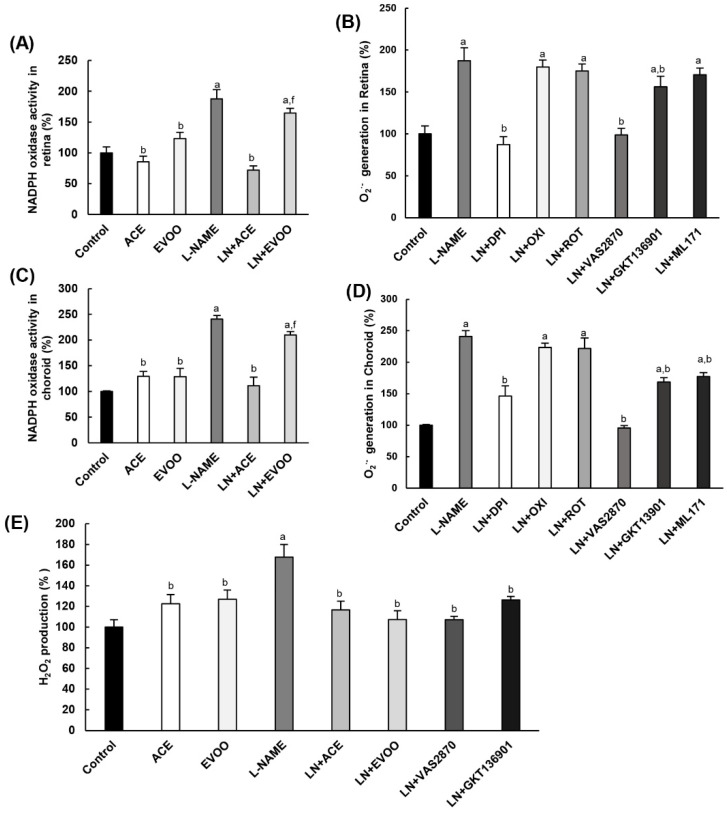
(**A**) Relative NADPH oxidase activity in retina homogenates and (**B**) characterization of the primary source of superoxide anion via preincubation with different inhibitors, as specified in Section 2.7. (**C**,**D**) Similar experiments as in (**A**,**B**) were performed in choroid homogenates. (**E**) H_2_O_2_ levels, measured by Amplex Red assay, in retina homogenates from all six experimental groups, including the effect of specific NOX inhibitors on L-NAME-treated animals. Values are expressed as mean ± SEM of four animals per group: ^a^ *p* < 0.05 vs. control; ^b^ *p* < 0.05 vs. L-NAME; ^f^ *p* < 0.05 vs. LN+ACE.

Since the NADPH oxidase system seems to play a major role in O_2_^•−^ production in retinal layers, the activity of this enzyme was determined in retina and choroid homogenates. In the retina, hypertensive mice showed a significant 1.9-fold increase relative to the control group, which was blocked by simultaneous administration of ACE oil (Figure 4A). Interestingly, such an effect was not mimicked by EVOO. Oil supplementation had no effect on normotensive (L-NAME-free) animals. As shown in Figure 4B, no changes were observed in retina samples from L-NAME mice following exposure to oxypurinol and rotenone, but DPI did restore O_2_^•−^ generation back to normal. Regarding more specific NOX inhibitors, preincubation with GKT136901 and ML171 reduced the excessive superoxide production in hypertensive animals only slightly (17% and 9%, respectively), while the use of VAS2870 resulted in a 99% reduction and led to values similar to those measured in the control group. A similar behavior was reproduced in choroid homogenates (Figure 4C,D). Therefore, not only is NADPH oxidase involved in the enhanced production of O_2_^•−^ in the retina and choroid of hypertensive animals, but NOX2 seems to be much more important for NADPH oxidase activation than NOX1 and NOX4 isoforms. No changes in O_2_^•−^. Production were observed in retinal and choroid samples from the control group in the presence and absence of NOX inhibitors (data not shown).

The Amplex Red assay in retina homogenates revealed excess H_2_O_2_ production (1.7-fold higher than normal) in L-NAME-treated mice. In this case, both ACE oil (117% of control) and EVOO (107%) were able to revert this H_2_O_2_ overproduction in hypertensive animals, leading to values similar to those returned by normotensive mice with or without dietary oil supplementation. Noteworthily, preincubation of retina homogenates from the L-NAME group with VAS2870 and GKT136901 also normalized H_2_O_2_ production (107% and 126%, respectively), thus confirming the major participation of NOX4 in H_2_O_2_ formation (Figure 4E). Again, H_2_O_2_ production was unaltered in retinal homogenates from the control group in the presence and absence of these NOX inhibitors (data not shown).

### 3.6. NOX Expression and Localization in Retinal Layers and Choroid

**Figure 5:**
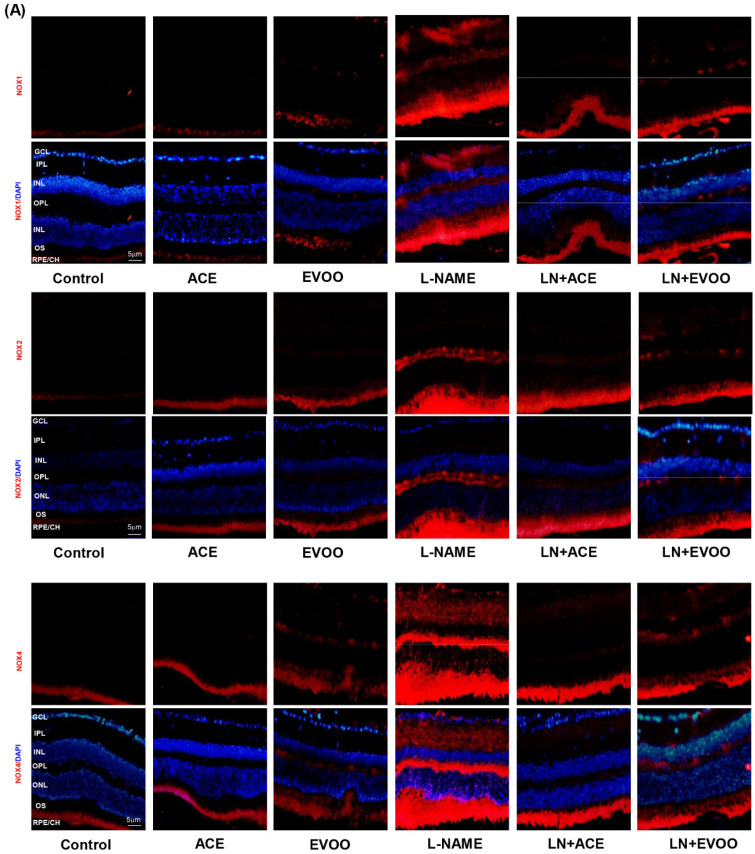

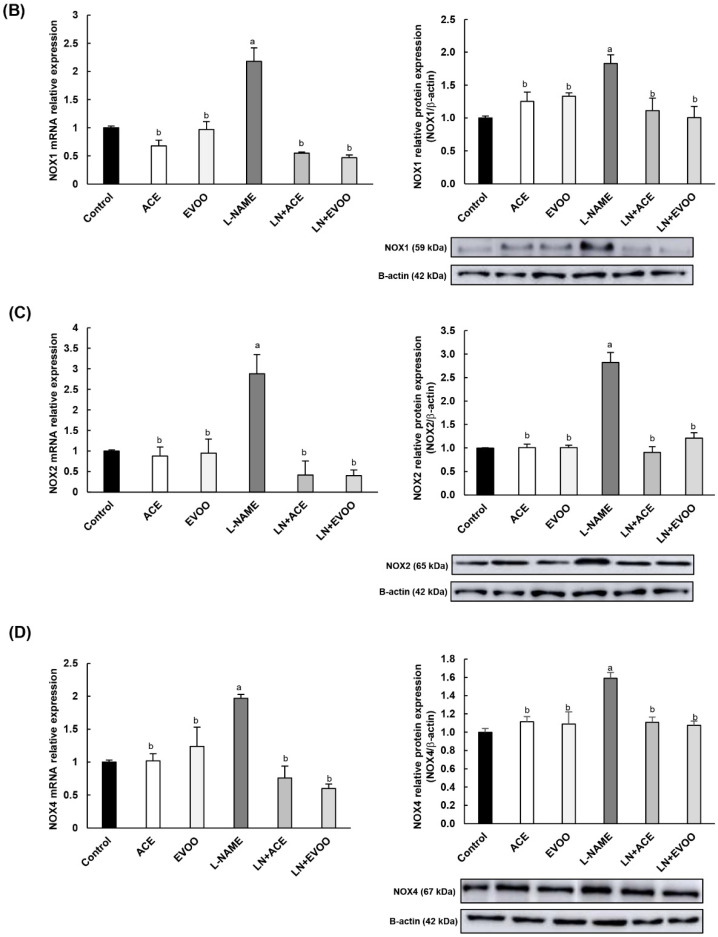
(**A**) NOX expression (red color) and double staining with DAPI (blue color) of NOX1 (top), NOX2 (middle) and NOX4 (bottom) in retinal layers from each experimental group. Magnification: 10×. mRNA and protein expression of (**B**) NOX1, (**C**) NOX2, and (**D**) NOX4 in retina homogenates from all groups. The quantitative fold changes in gene expression were determined relative to the corresponding value for the glyceraldehyde-3-phosphate dehydrogenase (GAPDH) housekeeping gene. Values are expressed as mean ± SEM of four animals per group: ^a^ *p* < 0.05 vs. control; ^b^ *p* < 0.05 vs. L-NAME. GCL: ganglion cell layer; IPL, inner plexiform layer; INL, inner nuclear layer; OPL, outer plexiform layer; ONL, outer nuclear layer; OS, outer segments; RPE/CH, retinal pigmentary epithelium/choroid.

NOX1, NOX2 and NOX4 immunofluorescence signals were significantly higher in L-NAME retinas in comparison with retinas from all other groups, including LN+ACE and LN+EVOO (Figure 5A). NOX1 and NOX4 were detected mainly in GCL, IPL, OPL, OS and RPE/CH, whereas NOX2 expression was only found in OPL, OS and RPE/CH. LN+ACE and LN+EVOO groups apparently showed a similar expression profile in the retina, except for a slightly higher NOX4 signal in the latter. Then, both mRNA and protein expression of NOXs isoforms were quantified in retinal homogenates and the results paralleled those observed in immunofluorescence experiments. An increase in mRNA and protein expression of NOX1 (2.18- and 1.83-fold change, respectively), NOX2 (2.88-/2.83-fold) and NOX4 (1.97-/1.59-fold) was found in retinas from the L-NAME-treated mice group in comparison with retinas from untreated animals (Figure 5B–D). ACE oil and EVOO-supplemented diets reduced the expression of all NOX isoforms in hypertensive animals to a similar extent back to normal values.

### 3.7. Nitric Oxide Synthase Expression, NO Concentration and Arginase Enzymes

**Figure 6:**
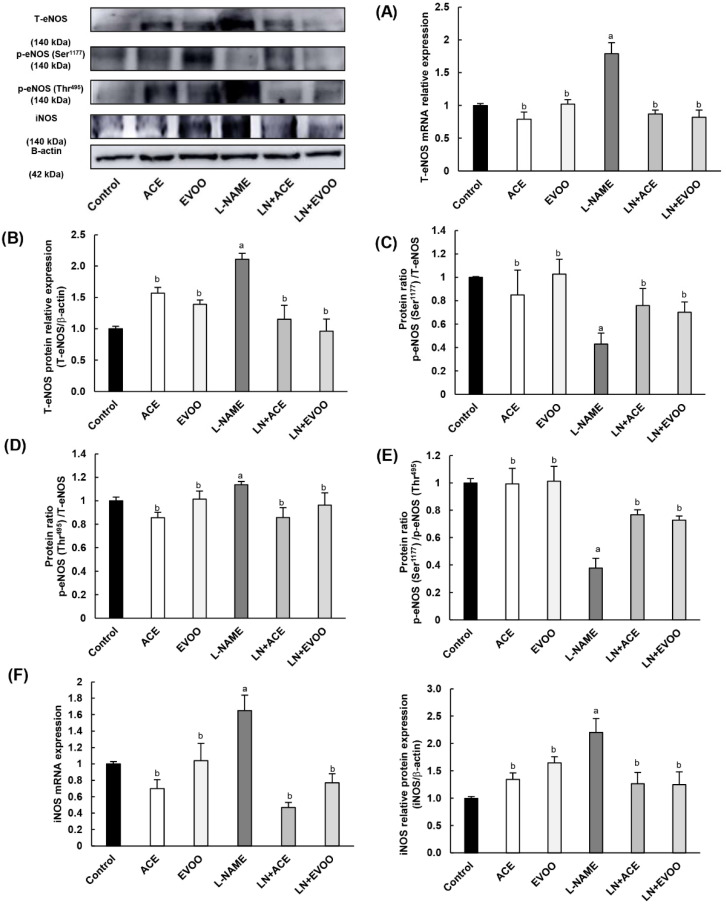

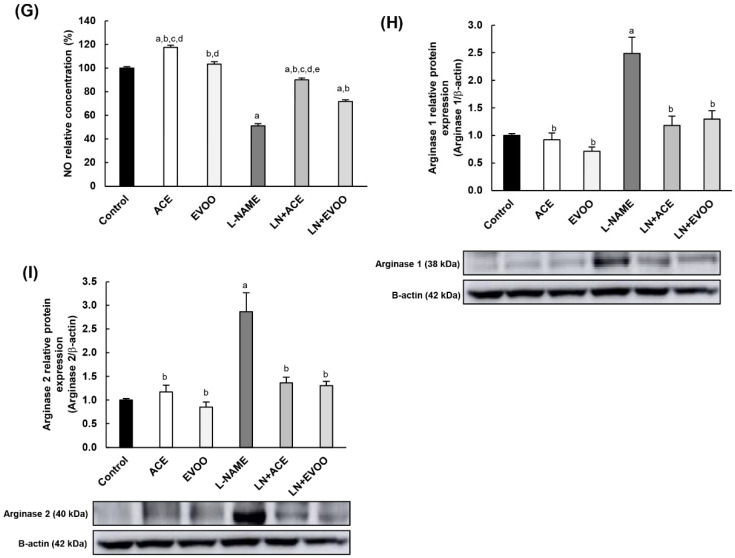

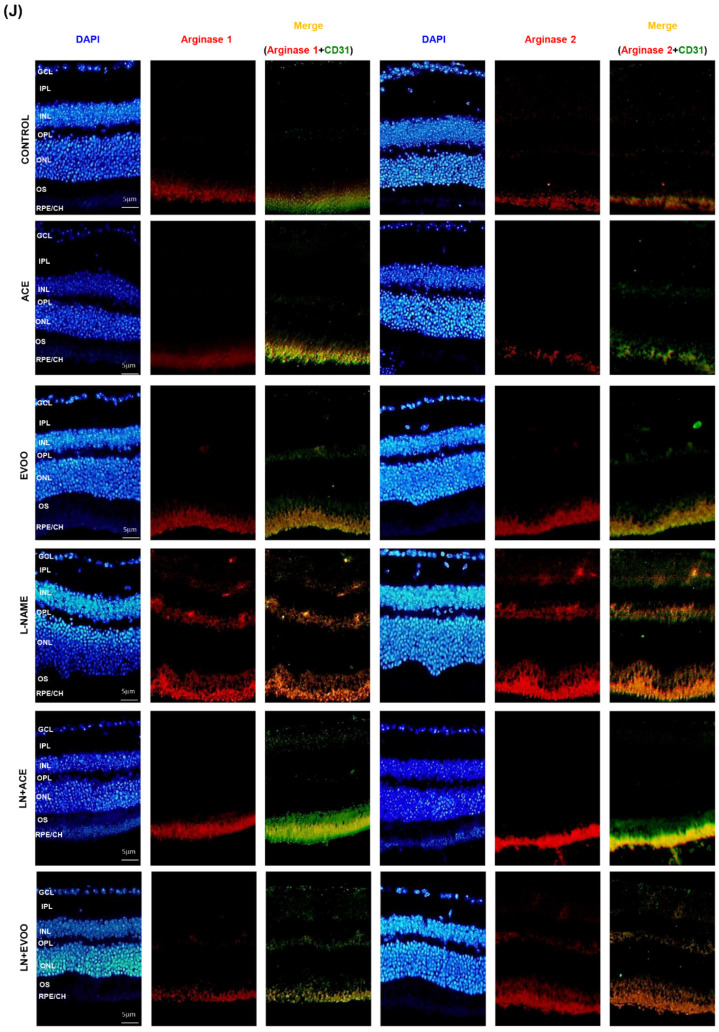
Total eNOS (T-eNOS) mRNA expression (**A**) and protein expression (**B**) in retina homogenates from all experimental groups. The activation status of eNOS enzyme was estimated from the ratios: (**C**) p-eNOS Ser^1177^/T-eNOS (activation); (**D**) p-eNOS Thr^495/^T-eNOS (inhibition); and (**E**) p-eNOS Ser^1177^/p-eNOS Thr^495^. (**F**) mRNA and protein expression of iNOS isoform in retina homogenates. (**G**) NO concentration in retina homogenates. (**H**,**I**) Protein expression of arginase isoforms 1 and 2 in retina homogenates. (**J**) Arginase 1 (left) and arginase 2 (right) expression (red color) and double staining with CD-31 (green color) in retinal layers, where the merge is represented in yellow color. Nuclei staining with DAPI (blue color) was used to identify retinal layer in each experimental group. Magnification: 10×. Values are expressed as mean ± SEM of four animals per group: ^a^ *p* < 0.05 vs. control; ^b^ *p* < 0.05 vs. L-NAME; ^c^ *p* < 0.05 vs. EVOO; ^d^ *p* < 0.05 vs. LN+EVOO; ^e^ *p* < 0.05 vs. ACE. GCL: ganglion cell layer; IPL, inner plexiform layer; INL, inner nuclear layer; OPL, outer plexiform layer; ONL, outer nuclear layer; OS, outer segments; RPE/CH, retinal pigmentary epithelium/choroid.

Experiments on NO metabolism and NO synthase (NOS) isoforms revealed a higher gene and protein expression of total endothelial isoform (T-eNOS) in the L-NAME group compared with all other experimental groups (Figure 6A,B). In particular, hypertensive mice showed a decrease in the phosphorylation of eNOS at Ser^1177^ (which reflects the activation of this enzyme), together with an increase in the phosphorylation at Thr^495^ (inhibitory phosphorylation) (Figure 6C,D). Consequently, a lower (0.63-fold) ratio of p-eNOS Ser^1177^/T-eNOS was found in this hypertensive model (Figure 6E). Both ACE oil- and EVOO-enriched diets lead to a similar increase in the ratio of p-eNOS Ser^1177^/p-eNOS Thr^495^. Non-hypertensive mice fed oil-enriched diets behaved similarly to the control group. Regarding the inducible (iNOS) isoform, NO-depleted mice showed a significant increase in mRNA and protein expression (1.65- and 2.2-fold change, respectively), which was also reversed by ACE oil and EVOO administration (Figure 6F).

NO concentration in retinal homogenates was reduced by 50% in L-NAME-treated animals; this alteration was corrected by oil supplementation, with a higher effect in favor of ACE oil-enriched diets compared to EVOO (Figure 6G). To further deepen NO metabolism, the location and protein expression of arginase enzyme isoforms 1 (Arg-1) and 2 (Arg-2), which are commissioned in L-arginine (NOS substrate) degradation, are depicted in Figure 6H–J. Both isoforms of this hydrolytic enzyme were overexpressed in the L-NAME group (2.42- and 2.87-fold change, respectively), and values returned to normal in both LN+ACE and LN+EVOO groups (Figure 6H,I). In addition, hypertensive animals presented with an overexpression of arginase isoforms in RPE/CH, OS, OPL and at the boundary GCL/IPL. Simultaneous dietary supplementation with oils revealed immunofluorescence signals in RPE/CH and OS, with a lower intensity than that observed in hypertensive mice subjected to the standard diet. In addition, LN+EVOO and EVOO groups showed a faint Arg-1/2 expression in OPL, an observation that was not reproduced in ACE or LN+ACE retinas. Experiments performed with an endothelial marker (anti-CD31) confirmed that arginase expression correlated with endothelial retinal cells, since the immunofluorescence signal from arginases merged with that from CD31 (yellow color in Figure 6J).

### 3.8. Antioxidant Enzymes and Transcription Factors in Retina Homogenates

**Figure 7:**
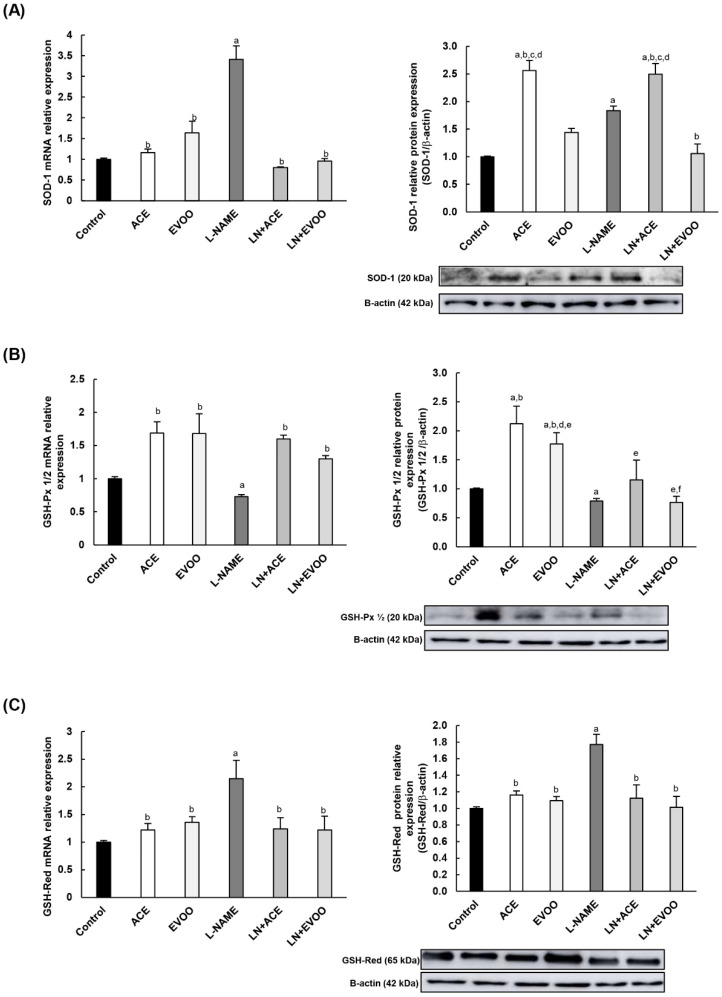

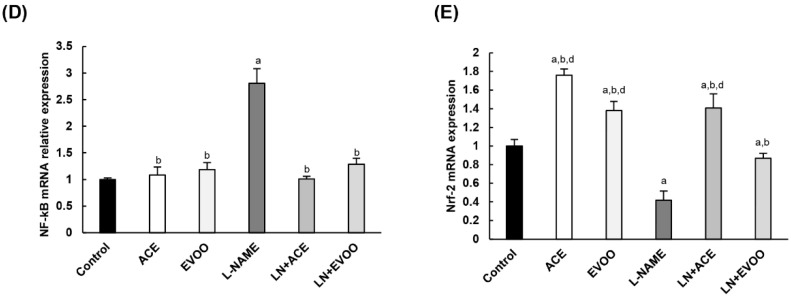
Antioxidant enzyme gene and protein expression of (**A**) superoxide dismutase (SOD-1), (**B**) glutathione peroxidase (GSH-Px) 1/2, and (**C**) glutathione reductase (GSH-Red) in retina homogenates from each experimental animal group. mRNA expression of transcription factors nuclear factor kappa-B (NF-kB) (**D**) and nuclear factor erythroid-2 (Nrf-2) (**E**) in the retina. The quantitative fold changes in gene expression were determined relative to GAPDH in each corresponding group. Values are expressed as mean ± SEM of four animals per group: ^a^ *p* < 0.05 vs. control; ^b^ *p* < 0.05 vs. L-NAME; ^c^ *p* < 0.05 vs. EVOO; ^d^ *p* < 0.05 vs. LN+EVOO; ^e^ *p* < 0.05 vs. ACE; ^f^ *p* < 0.05 vs. LN+ACE.

The antioxidant enzyme expression at mRNA and protein levels is shown in Figure 7. Both superoxide dismutase (SOD-1) and glutathione reductase (GSH-Red) displayed a significant upregulation (3.41- and 1.77-fold for SOD-1 gene/protein expression, respectively; and 2.15-/1.77-fold for GSH-Red) in mice subjected to L-NAME treatment compared to the control group (Figure 7A,C). Oil-enriched diets reversed these values back to those observed in control normotensive mice, with the sole exception of SOD-1 protein expression of in LN+ACE, in which values were almost 2.5 times higher than those observed in control animals. On the other hand, the glutathione peroxidase (GSH-Px) enzyme was downregulated in the L-NAME group at both mRNA (0.73-fold change) and protein (0.79-fold change) levels compared with the control group (Figure 7B), an alteration that was mitigated in hypertensive animals treated simultaneously with experimental oils.

Due the relevance of nuclear factor kappa-B (NF-kB) and nuclear factor erythroid-2 (Nrf-2) transcription factors as regulators of oxidative imbalance/inflammation related to NADPH oxidase and NOS pathways, their gene expression was also determined and measured by RT-qPCR. Overexpression of NF-kB (2.81-fold change over control group) was detected in the L-NAME group and reversed via simultaneous administration of oils (Figure 7D). On the contrary, Figure 7E depicts the observed downregulation of Nrf-2 in hypertensive animals (0.42-fold change), with a reversal action represented by ACE (1.76-fold over control group) and LN+ACE (1.41-fold) groups.

## 4. Discussion

Despite the well-known healthy effects of the Mediterranean diet and EVOO in particular, very little is known about the properties of other varieties of OOs, such as ACE (wild olive) oil. The chemical composition profile of the oils used in this study, i.e., ACE oil and EVOO (which were obtained from the same geographic area and processed following equal protocols) revealed a similar fatty acid composition, but interesting differences at the level of minor components. Thus, the unsaponifiable fraction from ACE oil was richer than that of EVOO in sterols, tocopherols, triterpene acids, alcohols and secoiridoids. Both sterols and tocopherols are well-known essential micronutrients in the diet of all mammals, with potent hypolipidemic and antioxidant capacities [49,50,51]. Furthermore, the proportion of triterpene acids in ACE is remarkable, considering the antioxidant and neuroprotective effects associated with maslinic acid, among others [52,53].

Despite the fact that the phenol content is similar in both oils, they showed an inverted ratio ortodiphenol/secoiridoids, the latter being the main polyphenols in ACE oil. A variety of pharmacological effects has been reported for these compounds against different pathologies related to inflammatory and oxidant events, due to their antidiabetic, antioxidant, anti-inflammatory, immunosuppressive, neuroprotective, anticancer, and anti-obesity properties [54,55,56,57]. Therefore, our findings on the chemical composition of ACE oil and EVOO suggest a different behavior in terms of health protection.

Experimental treatment with L-NAME is a well-established model of AH. The administration of oil-enriched diets and/or L-NAME did not affect food/water intake nor weight gain throughout the 6-week experimental period. As expected, a significant and sustained elevation of SBP and DBP was found in the L-NAME group [45]. Interestingly, hypertension was alleviated in L-NAME-treated animals subjected to simultaneous administration of ACE oil- and EVOO-enriched diets, but a clearly higher depletion of blood pressure was observed in the former. Olive polyphenols have been associated with positive blood pressure outcomes [58]; additional experiments carried out in our lab showed an improvement in endothelial function, vascular remodeling and hypertrophy in aortas from hypertensive animals upon administration of an ACE oil-enriched diet (unpublished results). Since no changes were found in total phenol content between both oils, the higher hypotensive effect observed for the ACE oil diet might perhaps be attributable to its elevated secoiridoid compound content.

No morphological changes in retinal layers nor signs of hypertension-induced cellular infiltration were revealed by hematoxylin–eosin staining. However, the morphometric analysis evidenced thinner GCL, OS and RPE/CH layers in the L-NAME group compared with normotensive animals. Similar results were reported in hypertensive patients without previous ocular abnormalities, which was associated with likely arterial sclerosis and vascular contraction due to a high intravascular pressure in the choroid [59], and a decrease in retinal blood flow [60]. These modifications in hypertensive eyes were reversed by the simultaneous administration of ACE oil and EVOO, suggesting a positive modulation of vascular sclerosis and retinal blood flow. Surprisingly, EVOO administration to normotensive animals also resulted in a decrease in OS and RPE/CH thickness when compared with control mice.

Oxidative stress is highly related to ocular pathologies, including AMD [61] or DR [31]; however, the origin of this oxidative imbalance and the pathways involved in the subsequent development of ocular damage are still under research. Preliminary experiments in our lab brought out an increase in ROS production and NADPH oxidase activity in retinas from L-NAME hypertensive Wistar rats (unpublished observations). In the current study, designed using L-NAME hypertensive C57B/6J mice, similar alterations were observed in both the retina and choroid layers of the eye. The increase in ROS generation in hypertensive mice could be reversed in all retinal layers by the simultaneous administration of ACE oil, whereas the EVOO-enriched diet only mitigated ROS overproduction in ONL and OS layers. These results might be attributable, at least in part, to the higher activity of the enzyme NADPH oxidase, an alteration that was also blocked in retina and choroid homogenates from the L-NAME+ACE group, but not in those from the L-NAME+EVOO group. Previous studies demonstrated a decrease in ROS production in LPS-induced murine peritoneal macrophages incubated with oleocanthal, one of the major secoiridoids present in OOs [62]. Therefore, the changes between ACE oil and EVOO might be due to a higher amount of secoiridoids in the former. Other bioactive minor compounds, such as tocopherols (also elevated in ACE oil over EVOO), might have contributed to better antioxidant outcomes for the ACE oil-enriched diet.

Studies using NOX inhibitors demonstrated a preferential role for the NOX2 isoform of NADPH oxidase in ROS production and NADPH oxidase activity in the retina and choroid of hypertensive animals, because the inhibitory action of GKT136901 and ML171 (affecting NOX1/NOX4) was much lower than that of VAS2870 (which can also inhibit NOX2). These results were confirmed by additional experiments on the gene and protein expression of NOXes, which revealed a significant rise in all three isoforms in the retinas of hypertensive mice, with NOX2 showing the highest upregulation. In this case, the simultaneous administration of both ACE oil and EVOO to L-NAME-treated animals brought the values back to levels observed in control animals. These results did not match those observed for NADPH oxidase activity, where the L-NAME+EVOO group retained the elevated values found in hypertensive mice; on the other hand, the increase reported in the L-NAME group for H2O2 production was reversed by the administration of either oils. Carnevale et al. [63] reported that EVOO downregulates NOX2 via H2O2, which is in agreement with the reduction in H2O2 found in the retinas of hypertensive mice fed either ACE oil or EVOO. Furthermore, it has been demonstrated that polyphenols from EVOO have effects on ROS levels and NOX expression [64,65]. Overall, these findings suggest that both oils (especially ACE oil) are able to counteract the hyperactivation of NADPH oxidase system with the subsequent regulation of ROS production, which eventually results in an improvement in AH-related retinal oxidative stress. This recovery of the oxidative balance was confirmed by the normalization of the protein expression of GFAP and nitrotyrosine as markers of microglial activation and oxidative stress in the retina. The downregulation of both parameters might indicate a better prognosis for some retinopathies, as previously reported for AMD and DR [61,66]. The effects of the oils in this regard might be attributed to minor components including hydroxytyrosol, as previously described [67].

Excessive ROS production and diminished NO bioavailability are associated with ocular pathologies [68,69]. The alterations observed in NO concentration and in the activation/expression of T-eNOS and iNOS in the retinas of hypertensive mice in our studies confirm the role of NO metabolism in eye diseases. Furthermore, the protein expression of arginase, an enzyme that competes with NOS for the use of the common substrate L-arginine, was significantly enhanced in L-NAME-treated animals. All these alterations were corrected by dietary administration of ACE oil and EVOO. The beneficial effects of the oils on NO metabolism might be due to the action of triterpene acids such as maslinic and oleanolic acids, which can activate eNOS via Ser1177 phosphorylation and increase NO production [70,71], and also to oleocanthal, which has previously been reported to downregulate iNOS expression [62]. eNOS activation is considered beneficial in some retinopathies [72], whereas iNOS is usually considered a biomarker of oxidative stress and inflammation in retinopathies such as AMD [73] or DR [74]. In the current study, both ACE oil and EVOO were able to reduce iNOS expression in hypertensive animals back to normal values, thus suggesting an additional protective effect against AH-associated retinal damage.

There is a great deal of evidence concerning the role of arginase in the regulation of NOS system, especially in the context of hypertension [75]. Two isoforms of arginase enzymes (Arg-1 and, in a major proportion, Arg-2) are expressed in the retina [76], whose regulation could be crucial in the development of some retinopathies such as DR [76] or retinal ischemia [77]. An upregulation of these isoforms correlates positively with the development of ocular pathologies, while Arg-2 depletion showed neuroprotective effects in optic nerve trauma [78] and in hyperoxia-induced retinal vascular degeneration [79]. In fact, the inhibition of Arg-1 activity has been suggested as a possible therapeutic strategy to alleviate DR [77].

Rojas et al. [80] showed that diabetes-induced endothelial cell senescence was due to NOX2 activation and subsequent ROS production, leading to an increase in arginase expression/activity that, in turn, led to a reduction in NO in the retina and favored eNOS uncoupling. Retinas from our animal model of L-NAME-induced hypertension seem to follow a mechanism similar to that described in diabetic retinas. Therefore, the association between NOX2 and arginase downregulation found in the retina after the administration of ACE oil and EVOO confers to these oils a retinoprotective effect in the hypertensive context.

Concerning the protein and gene expression of antioxidant enzymes, SOD-1 and GSH-Red were upregulated in retinas from L-NAME-treated animals, whereas the opposite pattern was observed for the GSH-Px enzyme. Simultaneous administration with oils reversed these alterations and led to values similar to those observed in normotensive mice. Interestingly, a higher protein expression of SOD-1 was found in ACE oil groups when compared with the equivalent EVOO groups. We hypothesize that the strong effect of ACE oil on SOD expression favors the conversion of O_2_^•−^ to H2O2, and excess H2O2 is then reduced by GSH-Px into H_2_O and O_2_ at the expense of GSH-Red. This hypothesis is confirmed by the above results, showing a reduction in O_2_^•−^, H_2_O_2_ and nitrotyrosine levels in ACE oil-fed hypertensive mice. Our results are in agreement with previous studies reporting that the secoiridoid oleuropein, a major component of olive polyphenols, increased the levels of SOD and GSH-Px in gentamicin-induced renal toxicity and in cisplatin-induced renal injury models [62,81,82], as well as in the kidneys of rats with unilateral ureteral obstruction [83]. However, due to the complexity of the biochemical pathways involved and the relevance of antioxidant enzymes in the retina, their implication in the eye is currently unpredictable [84], and different antioxidant profiles are found depending on the specific retinopathy, as reported in AMD [85] and DR [86], among others.

In addition to studying the NADPH oxidase system, NO metabolism, GFAP expression and antioxidant enzyme profile, we finally explore the modulation of transcription factors NF-κB and Nrf-2 by ACE oil and EVOO. NF-κB is a well-known regulator of inflammation and oxidative processes surrounding cardiovascular diseases and hypertension [87]; it has been implicated in different retinopathies, such as hypertensive retinopathy induced by angiotensin [88], AMD [89] and DR [90]. Our data demonstrated an overexpression of NF-κB in hypertensive L-NAME retinas that was reverted by ACE oil- and EVOO-enriched diets. EVOO has classically been attributed as having anti-inflammatory actions [91], mainly due to its minor polyphenol constituents [11], which can affect the expression of NF-κB [90]. The high content of triterpene acids in ACE oil might also be implicated in this effect. Additionally, Ampofo et al. [92] described a maslinic acid-dependent downregulation of NF-κB in endothelial cells, paralleled by increased eNOS expression and reduced oxidative DNA damage. Experiments carried out in human umbilical vein endothelial cells (HUVEC) also demonstrated NF-κB downregulation by maslinic acid [93]. Furthermore, ursolic and oleanolic acids have also been described as promising anti-inflammatory compounds via NF-κB inactivation [94].

Nrf-2 is a transcription factor that activates important cellular defense mechanisms against oxidative stress and is associated with neuroprotective mechanisms [95]. Several authors have focused on the role of Nrf-2 in retinal diseases in which this factor modulates antioxidant pathways, such as in AMD [96], DR [97], or ischemic retinopathy, where a novel therapy based on Nrf-2 activation was able to counteract the oxidative retinal damage [98]. In this sense, our results demonstrated that ACE oil and EVOO upregulated retinal Nrf-2 expression, with a preferential effect in favor of the former. Several authors have attributed to OO the ability to modulate Nrf-2 [99], where polyphenols such as secoiridoids seem to be positively implicated in this effect [62], as these are also associated with a reduction in NF-κB levels [100]. In the same way, maslinic [93] and ursolic acids [101] are also positive modulators of Nrf-2 with beneficial effects against oxidative/inflammatory damage in different situations.

Despite the fact that our data point out the retinoprotective effect of ACE oil and EVOO on hypertensive mice based upon their antioxidant capacity, the wide variability of components obtained from any oil extraction warrants further studies using more specific components, in order to clarify their role in the different molecular pathways affected by oil diets. Moreover, specific chemical analysis should be considered with oil extracts obtained from different geographic areas and subjected to additional extraction methods. Finally, due to the limited number of animals used in the current study, subsequent in vivo and in vitro experiments will certainly shed light on the intracellular pathways modulated by ACE oil and eventually help explore its function in human trials.

## 5. Conclusions

In conclusion, our study demonstrates the presence of increased oxidative stress and inflammation in the choroid and retina in a mouse model of L-NAME-induced hypertension. More importantly, we show—for the first time—that ACE (wild olive) oil showed a greater ability to counteract the pathogenic mechanisms of hypertensive eye diseases when compared with a reference EVOO of a similar geographic origin, including systemic anti-hypertensive effects and a significant improvement in hypertension-related oxidative retinal damage. These better outcomes in favor of ACE oil might be attributable to a higher content of sterols, tocopherols, triterpenes and secoiridoids in comparison with EVOO. We claim that the rational use of ACE oil-enriched diets can offer a novel retinoprotective strategy to neutralize AH-related retinal oxidative damage and associated pathologies, including AMD, hypertensive retinopathy, glaucoma and other retinopathies, as well as choroidal vascular diseases.

## 6. Patents

The authors applied for a patent relating to the retinoprotective effect of acebuche oil (application number P202030625) on 23 June 2020 at the Oficina Española de Patentes y Marcas (OEPM), Ministerio de Industria, Comercio y Turismo. The authors confirm that these results have not been published to date.

## Figures and Tables

**Table 1:** Content of fatty acids, sterols, tocopherols, pentacyclic triterpenes and polyphenols in extra virgin olive oil (EVOO) and acebuche (ACE) oil.

Class	Compound	Extra Virgin Olive Oil (EVOO)	Acebuchina Oil (ACE Oil)
**Fatty acids (%)**	Myristic acid. C14:0	<LOD ^1^	<LOD
	Palmitic acid. C16:0	10.56	12.90
	Palmitoleic acid. C16:1	0.79	1.25
	Margaric acid. C17:0	0.11	0.09
	Margaroleic acid. C17:1	0.17	0.16
	Stearic acid. C18:0	2.97	2.36
	Oleic acid. C18:1	76.61	74.42
	Linoleic acid. C18:2	7.18	7.34
	Arachidonic acid. C20:0	0.44	0.39
	Linolenic acid. C18:3	0.77	0.70
	Gondoic acid. C20:1	0.31	0.29
	Behenic acid. C22:0	0.11	0.12
	Lignoceric acid. C24:0	<LOD	<LOD
	**Total (%)**	**100.0**	**100.0**
	Acidity (%C18:1)	≤ 0.8	0.14
			
**Sterols (%)**	Cholesterol	0.36	0.37
	Bassicasterol	<LOD	<LOD
	24-Methylenecholesterol	<LOD	<LOD
	Campesterol	3.06	3.59
	Campestanol	<LOD	<LOD
	Stigmasterol	0.59	0.96
	Δ7-Campesterol	<LOD	<LOD
	Δ5,23-Stigmastadienol	<LOD	<LOD
	Clerosterol	1.07	1.02
	Δ-Sitosterol	84.60	85.89
	Sitostanol	1.46	1.1
	Δ5-Avenasterol	7.44	7.89
	Δ5,24-Stigmastadienol	0.37	0.64
	Δ7-Stigmastenol	0.31	0.25
	Δ7-Avenasterol	0.49	0.46
	**Total (mg/kg)**	**1531.4**	**1735**
			
**Tocopherols (%)**	α-Tocopherol	94.60	97.2
	β-Tocopherol	2.4	0.8
	γ-Tocopherol	3.0	2.1
	δ-Tocopherol	<LOD	<LOD
	**Total (mg/kg)**	**221.76**	**343.8**
**Pentacyclic triterpenes**			
Triterpene acids (%)	Oleanolic acid	27.03	34.5
	Ursolic acid	37.93	24.9
	Maslinic acid	34.46	40.6
	**Total (mg/kg)**	**153.12**	**340.3**
Triterpene alcohols	Erythrodiol+Uvaol	37	45.6
(mg/kg)			
**Polyphenols**	Ortodiphenols	125	86.7
(mg/kg)	Secoiridoids	83	147.8
	**Total (mg/kg)**	**250**	**261.8**
	**Total (mg/kg in Tyrosol)**	**162**	**170**

^1^ LOD, limit of detection.

**Table 2:** Antibodies used for immunofluorescence studies.

Primary Antibody	Origin	Dilution	Reference
Anti-NOX1	Mouse monoclonal	1:200	Santa Cruz Biotechnology, Santa Cruz, CA, USA
Anti-NOX2	Rabbit monoclonal	1:100	Epitomics-Abcam, Burlingame, CA, USA
Anti-NOX4	Rabbit monoclonal	1:500	Epitomics-Abcam
Anti-GFAP	Mouse monoclonal	1:500	Santa Cruz Biotechnology
Anti-Arginase 1	Mouse monoclonal	1:100	Santa Cruz Biotechnology
Anti-Arginase 2	Mouse monoclonal	1:100	Santa Cruz Biotechnology
Anti-CD31	Rabbit monoclonal	1:200	Rockland Immunochemicals, Limerick, PA, USA

**Table 3:** Antibodies used for Western blotting analysis.

Primary Antibody	Origin	Dilution	Secondary Antibody	Dilution	Reference
Anti-NOX1	Mouse monoclonal	1:1000	Goat Anti-Mouse	1:2000	Santa Cruz Biotechnology, CA, USA
Anti-NOX2	Rabbit monoclonal	1:8000	Goat Anti-Rabbit	1:9000	Epitomics-Abcam, Burlingame, CA, USA
Anti-NOX4	Rabbit monoclonal	1:7000	Goat Anti-Rabbit	1:8000	Epitomics-Abcam
Anti-T-eNOS	Mouse monoclonal	1:1000	Goat Anti-Mouse	1:2000	Santa Cruz Biotechnology
Anti-p-eNOS Ser^1177^	Mouse monoclonal	1:1000	Goat Anti-Mouse	1:2000	Santa Cruz Biotechnology
Anti-p-eNOS Thr^495^	Mouse monoclonal	1:1000	Goat Anti-Mouse	1:2000	Santa Cruz Biotechnology
Anti-iNOS	Mouse monoclonal	1:1000	Goat Anti-Mouse	1:2000	Santa Cruz Biotechnology
Anti-Nitrotyrosine	Mouse Monoclonal	1:1000	Goat Anti-Mouse	1:2000	Santa Cruz Biotechnology
Anti-GFAP	Mouse monoclonal	1:2000	Goat Anti-Mouse	1:4000	Santa Cruz Biotechnology
Anti-GSH-Px1/2	Mouse monoclonal	1:1000	Goat Anti-Mouse	1:4000	Santa Cruz Biotechnology
Anti-GSH-Red	Rabbit polyclonal	1:5000	Goat Anti-Rabbit	1:8000	Santa Cruz Biotechnology
Anti-SOD-1	Mouse monoclonal	1:1000	Goat Anti-Mouse	1:2000	Santa Cruz Biotechnology
Anti-Arginase 1	Mouse monoclonal	1:1000	Goat Anti-Mouse	1:2000	Santa Cruz Biotechnology
Anti-Arginase 2	Mouse monoclonal	1:1000	Goat Anti-Mouse	1:2000	Santa Cruz Biotechnology
Anti-β-Actin	Mouse monoclonal	1:20,000	Goat Anti-Mouse	1:30,000	Santa Cruz Biotechnology

**Table 4:** Primers used for real-time PCR.

Gene	Forward Primer (5′→3′)	Reverse Primer (5′→3′)	Accesion Number
NOX1	TTCACCAATTCCCAGGATTGAAGTGGATGGTC	GACCTGTCACGATGTCAGTGGCCTTGTCAA	AY174116.1
NOX2	CCCTTTGGTACAGCCAGTGAAGAT	CAATCCCACGTCCCACTAACATCA	FJ168469.1
NOX4	ATCACAGAAGGTCCCTAGCA	TAACCATGAGGAACAATACCAC	AF276957.1
eNOS	AACTCCTGTCTTCCATCAAGAG	TTCACTGCATTGGCTACTTCC	U53142.1
iNOS	TTTGTGCGAAGTGTCAGTGG	CCTCCTTTGAGCCCTTTGTG	BC062378.1
GSH-Px1/2	GGAGAATGGCAAGAATGAAGA	CCGCAGGAAGGTAAAGAG	NM001329528.1
GSH-Red	CACCTCTTCCTTCGACTACC	GCTTGATGACATGCCAACTG	BC056358.1
SOD-1	CGTCATTCACTTCGAGCAGAAGG	GTCTGAGACTCAGACCACATA	AF223251.1
NF-κB	CCCTAAAGATTGTGCCAAGAG	GAAAGAGGTTATCCTGAAATCCC	BC138535.1
Nrf-2	ACATTCCCAAACAAGATGCC	GGTATTAAGACACTGTAATTCGGG	BC026943.1
GAPDH	GCCAAAAGGGTCATCATCTCCGC	GGATGACCTTGCCCACAGCCTTG	XM017321385.2
